# Preparation of nanostructured photocatalyst ZnSnO_3_@S-doped g-C_3_N_4_ and its use in DB1 dye degradation through photocatalytic ozonation process

**DOI:** 10.1016/j.heliyon.2024.e25451

**Published:** 2024-02-01

**Authors:** Elaheh Bahadorirad, Shahab Maghsoudi, Elham Jalali

**Affiliations:** a Department of Chemistry, Shahid Bahonar University of Kerman, Kerman, Iran, P.O. Box 76169-133, Kerman, Iran; b Young Researchers Society, Shahid Bahonar University of Kerman, Kerman, Iran, P.O. Box 76175-133, Kerman, Iran

**Keywords:** Photocatalytic ozonation process (PCO), DB1, ZnSnO_3_@S-doped g-C_3_N_4_, Photodegradation

## Abstract

This study aimed to investigate the potential of the photocatalytic ozonation process (PCO) for decolorizing DB1(direct blue) dye, a commonly used dye in the textile industry known for its resistance to removal from wastewater. To address this challenge, a ZnSnO_3_@S-doped g-C_3_N_4_ nano photocatalyst was synthesized using a simple hydrothermal method. In a novel approach, a light/O_3_/ZnSnO_3_@S-doped g-C_3_N_4_ system was employed for the first time to degrade the DB1 dye. BET analysis indicated that the synthesized catalyst exhibited the fifth type of isotherm, typically associated with materials containing mesopores. Under optimized conditions, the PCO process achieved complete decolorization of 70 ppm DB1 dye within just 15 min at a temperature of 25 °C, a gas flow rate of 2.83 ml/s, and a catalyst dosage of 0.003 g, encompassing both removal and photocatalytic contributions. Importantly, the catalyst demonstrated excellent stability and could be reused up to five times. These findings highlight the promising potential of the light/O_3_/ZnSnO_3_@S-doped g-C_3_N_4_ system in effectively decolorizing DB1 dye, overcoming its resistance, and addressing an important challenge faced by the textile industry in wastewater treatment. The formative nature of this study provides valuable insights into the development of advanced oxidation processes for efficient dye removal.

## Highlights

• First use of photocatalytic ozonation ZnSnO_3_@S-doped g-C_3_N_4 for DB1 degradation._
• Synergistic effects with ozone when combined with ZnSnO_3_@S-doped g-C_3_N_4_ nano photocatalyst
• Stable ZnSnO_3_@S-doped g-C_3_N_4_ nano photocatalyst for water treatment, reusable up to five times
• Rapid decomposition of dye molecules into smaller, less harmful substances.

## 1 Introduction

**Fig. 1:**
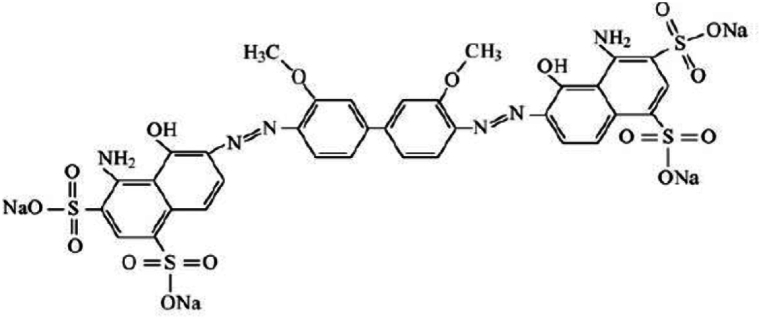
Structure of DB1 dye [8].

Textile dyeing accounts for 17 to 20 percent of textile wastewater [1]. Each year, these industries release large amounts of colored wastewater into the environment. As a result of fabrics' low dye absorption, they must apply a large amount of dye [2]. As azo dyes are highly resistant to washing and light, they are overused in textile manufacturing. Due to their high usage, they comprise 70 % of the dyes used in these sectors [3]. Azo dyes cause environmental problems and health risks such as disturbance of photosynthesis of aquatic plants [4], soil eutrophication [5], ecosystem changes [6], toxic amine production, and consequently cancer [7]. The DB1 dye (Fig. 1) has a very high molecular weight (992.82 g/mol) [8] and is resistant to temperatures above 300 °C, making it difficult to decolorize [9].

Advanced oxidation processes (AOPs) are a useful method for the degradation of dyes. In the last two decades, researchers have studied the effect of photocatalytic ozonation on the degradation of Orange II Dye utilizing Bi_2_O_3_ and Au/Bi_2_O_3_ nanorods [10] azo dye Acid Blue 113 using Fe_2_O_3_/MoS_2_ nanocomposite [11], and organic contaminants in seawater with TiO_2_ [12]. The oxidizing power of ozone makes it an excellent replacement for oxygen in the oxidation process. The light/O_3_/photocatalyst system is not only economical but also highly efficient compared to each of these processes singly [12]. Research shows that to produce each hydroxyl radical, three electrons must be trapped when O_2_ is used as an electron acceptor. When O_3_ is the electron acceptor, one electron is enough to produce one hydroxyl radical. ZnSnO_3_ is one of the promising compounds that has been used successfully in photocatalytic reactions to degrade dyes such as Rhodamine B [13], Methyl blue, Indigo carmine, and Acid violet [14]. There has always been interest in graphite carbon nitride (g-C_3_N_4_) due to its compatibility with the environment, its low cost, stable and unique properties. There is, however, the possibility of electron-hole pairs recombination in both ZnSnO_3_ and g-C_3_N_4_ when used separately ([[15], [16], [17]]). This, in turn, can lead to a lower pollutant degradation rate. Researchers have improved the performance of photocatalysts by engineering their surfaces. In terms of pollutant degradation, the hybrid Bi_2_O_3_/g-C_3_N_4_ composite showed effective results [18]. Photocatalytic degradation of methylene blue under sunlight was also studied by the g-C_3_N_4_@BiOCl composite [19], and the ternary composite of PANI/BiOCl/GO showed favorable results in photocatalytic mineralization of Rhb dye [20]. Sulfur doping of g-C_3_N_4_ can enhance its efficiency in the photocatalytic reaction as a cost-effective and simple method of changing its electronic structure [21]. Doping g-C_3_N_4_ with sulfur can increase its photocatalytic activity because impurities on the surface separate electron-hole pairs, reduce recombination, and boost efficiency [22]. During the photocatalytic reaction, ZnSnO_3_ particles tend to aggregate [15,17]. Therefore, creating a composite of ZnSnO_3_ and g-C_3_N_4_ and connecting them heterogeneously also produced excellent charge separation. Combining ZnSnO_3_ with g-C_3_N_4_ and forming composites with ZnSnO_3_@g-C_3_N_4_ has shown effective results in the photocatalytic degradation of tetracycline [23].

In the present work, S-doped g-C_3_N_4_ was first prepared. A simple hydrothermal process was then used to synthesize ZnSnO_3_@S-doped g-C_3_N_4_ nanocomposite for the first time. After characterization, the degradation of DB1 dye was carried out through a photocatalytic ozonation process. A TOC test was conducted to determine mineralization. A real sample was also simulated to check the effectiveness of the test under real conditions, and successful results were obtained.

## 2 Experimental

### 2.1 Materials

Thiourea, zinc acetate (Zn(CH₃CO₂)₂·2H₂O), tin(IV) chloride pentahydrate (SnCl_4_·5H_2_O), sodium hydroxide (NaOH), and sulfuric acid (H_2_SO_4_) were purchased from Merck (Germany). DB1 dye was purchased from Tianjin China Co. Ozone gas was produced by an ozone generator with a capacity of 5 ml/s and a power of 66 W of Bootiatech Iran.

### 2.2 Preparation of S-doped g-C_3_N_4_

The sulfur doping in g-C_3_N_4_ was achieved by using thiourea as the sulfur source during the synthesis process. Briefly, 10g of thiourea was grinned perfectly. It is then transferred into a crucible and heated for 4 h at 550 °C with a ramp rate of 300 °C/h. After cooling to room temperature, the obtained yellow solid substance was thoroughly ground in a mortar and washed several times with distilled water. In the following step, it was placed in a vacuum oven at 60 °C for 15 h to dry [21,24]. It is stored as bulk S-doped g-C_3_N_4_.

To synthesize nanosheet S-doped g-C_3_N_4_ from bulk form, 0.3 g of the bulk g-C_3_N_4_ was mixed with 10 ml of methanol and sonicated for 2 h. Then, it was washed with distilled water and centrifuged. After drying in a vacuum dryer at 60 °C for 15 h, S-doped g-C_3_N_4_ nanosheets were obtained [24].

### 2.3 Preparation of ZnSnO_3_@S-doped g-C_3_N_4_ nanocomposite

A certain amount of S-doped g-C_3_N_4_ powder was added to a solution consisting of 0.0296 g Zn(CH₃CO₂)₂·2H₂O and 0.0473 g SnCl_4_.5H_2_O and 44 ml NaOH (0.2 M). The solution was stirred and sonicated for 1 h. It was then transferred into a 60 ml Teflon-lined stainless steel autoclave and maintained at 160 °C for 11 h. After cooling to room temperature, precipitates were gathered and washed with methanol and deionized water several times to prepare the pure product. This was followed by 7 h of drying at 90 °C. As a way of investigating the effect of the ratios between ZnSnO_3_ and S-doped g-C_3_N_4_ on dye degradation, the nanocomposite was synthesized with different mass fractions of zinc acetate (7.5 %, 10 %, 12.5 %, and 15 %) [23].

### 2.4 Photocatalytic ozonation process measurement

**Scheme 1:**
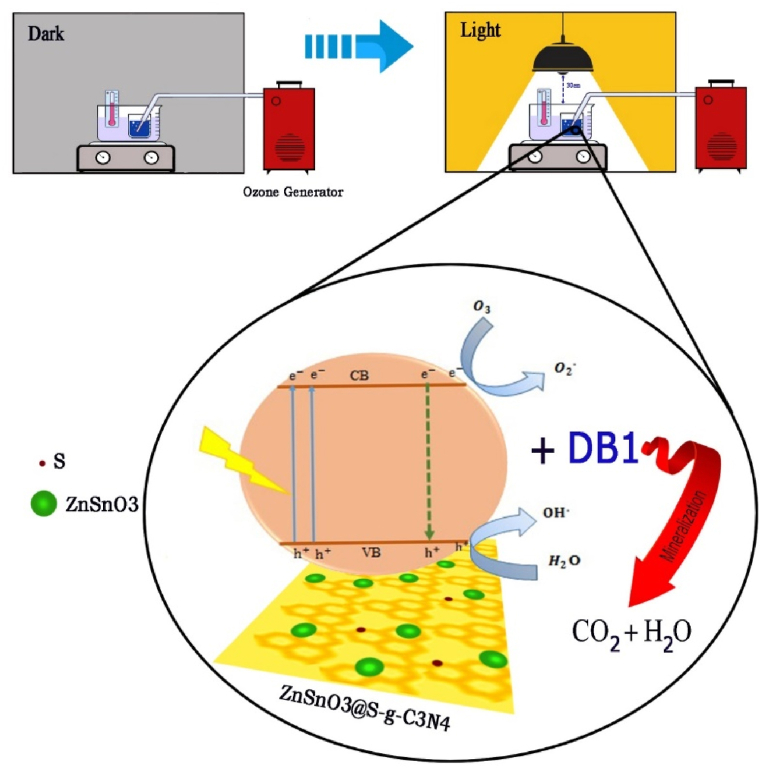
Schematic illustration of DB1 dye degradation through a photocatalytic ozonation process.

To evaluate the activity of nanocomposite during the photocatalytic ozonation process for DB1 dye degradation, 0.0075 g of ZnSnO_3_@S-doped g-C_3_N_4_ was added to a 15 mL 50 ppm solution of dye (pH 5). During the entire process, the tube connected to the ozone generator was adjusted so that ozone gas entered the solution continuously with a flow rate of 2.83 ml/s. At first, to reach adsorption equilibrium, the solution was stirred for 15 min in the dark. Then a specific amount of homogeneous solution was gathered. Afterwards, it was exposed to a 50W lamp (at a distance of 20 cm from the top) for 15 min to achieve a photocatalytic reaction. A UV–vis spectrophotometer was used to measure the absorbance of the desired solution at 661 nm (Scheme 1).

To calculate the percentage of photocatalytic degradation of DB1 dye considering the effect of adsorption equation (1) was used.

(1) $$\text{Degradation}\;\text{efficiency}=\left(C_{D}‐C_{L}\right)\;/\;\left(C_{S}‐C_{D}\right)\times 100$$

Where C_L_, C_D,_ and C_S_ are the concentration of DB1 (after exposure), sample (after surface adsorption in the absence of light), and stock sample, respectively.

The used photocatalyst was separated from the solution using a centrifuge, washed well with distilled water and methanol, and reused.

## 3 Results and discussion

### 3.1 Characterization

**Fig. 2:**
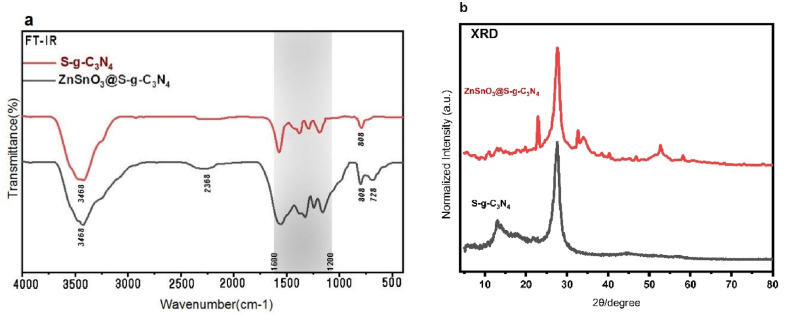
FTIR spectra of **(a)** ZnSnO_3_@S-doped g-C_3_N_4_ and S-doped g-C_3_N_4_, and **(b)** XRD pattern for ZnSnO_3_@S-doped g-C_3_N_4_ and S-doped g-C_3_N_4_.

The FT-IR spectrum of S-doped g-C_3_N_4_ is shown in Fig. 2 (a). wide peak around 3500 cm^−1^ to 3000 cm^−1^ is related to water molecules absorbed by the surface. Also, it can point to stretching vibrations of N–H of amine groups [25]. Peaks at regions of 1200 cm^−1^ to 1600 cm^−1^ correspond to the stretching vibrations of C=N and C–C bonds of g-C_3_N_4_. Also, a peak at a wave number of 808 cm^−1^ is related to the vibration of characteristic triazine [26]. ZnSnO_3_@S-doped g-C_3_N_4_ nanocomposite FT-IR spectrum is also shown in Fig. 2 (a). Peaks at 3468 cm^−1^, 2368 cm^−1^ and 728 cm^−1^ confirm the Sn–O and ZnO bonds in ZnSnO_3_ [27].

An X-ray diffraction pattern of S-doped g-C_3_N_4_ is shown in Fig. 2(b). According to the figure, two distinct peaks are found at the 2θ range of 12.96° and 27.18°, which correspond to X-rays incident on (002) and (100) planes, respectively, and match the standard (JCPS-87-1526) pattern of graphitic carbon nitride doped with sulfur [28]. Moreover, the X-ray diffraction analysis shown in Fig. 2 b reveals characteristic peaks at 22.9°, 32.98°, 38.8°, 47°, and 58.5° that match the standard pattern (JCPDS-11-0274) associated with ZnSnO_3_. This observation provides evidence that ZnSnO_3_ is present in the ZnSnO_3_@S-doped g-C_3_N_4_ nanocomposite synthesized [29].

**Fig. 3:**
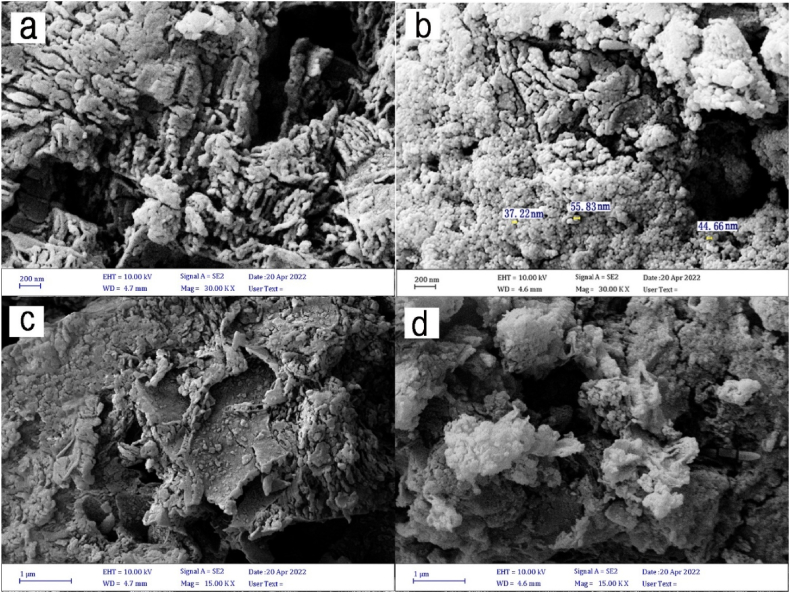
**(a)** SEM images of S-doped g-C_3_N_4_, **(b)** and ZnSnO_3_@S-doped g-C_3_N_4_**, (c)** SEM images of S-doped g-C_3_N_4_ (1 μm**)**, **(d)** and ZnSnO_3_@S-doped g-C_3_N_4_ (1 μm).

SEM images of graphitic carbon nitride doped with sulfur are shown in Fig. 3 (a and c), and ZnSnO_3_@S-doped g-C_3_N_4_ is shown in Fig. 3 (b and d). As can be seen, these nanoplates have a two-dimensional structure. The morphology and size of the nanocomposite particles are shown in Fig. 3(b). Compared to graphitic carbon nitride nanosheets alone, the change in the surface morphology of S-doped g-C_3_N_4_ nanosheets after combination with ZnSnO_3_ is evident. On S-doped g-C_3_N_4_ sheets, ZnSnO_3_ particles are well dispersed. The average particle size of it is around 45 nm.

**Fig. 4:**
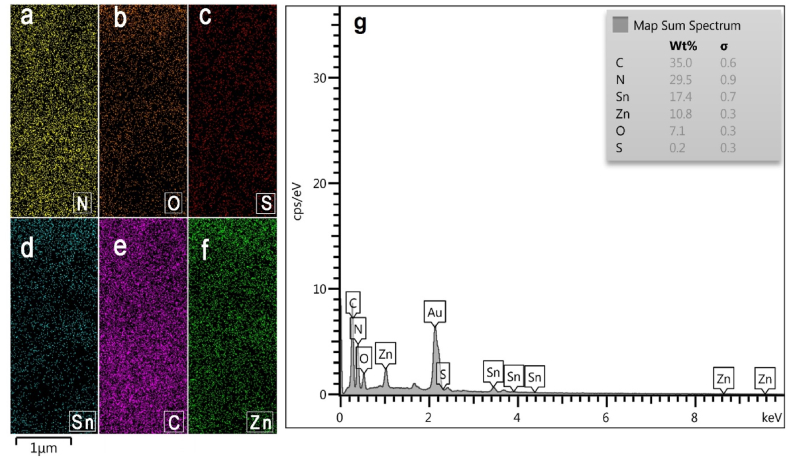
**)a, b, c, d, e and f(**the EDX mapping analysis of ZnSnO_3_@S-doped g-C_3_N_4_ that shows the elemental distribution of N, O, S, Sn, C, and Zn respectively, and **(g)** the percentage of them in a ZnSnO_3_@S-doped g-C_3_N_4_ sample.

It is possible to determine the type and percentage of elements in a sample (Fig. 4 g) using highly advanced Energy Dispersive X-ray analysis (EDX). A Low amount of sulfur doping in ZnSnO_3_@S-doped g-C_3_N_4_ could potentially be challenging to detect using conventional characterization techniques such as SEM and EDX. The low concentration of sulfur, coupled with the inherent limitations of EDX, could lead to undetectable or weak signals. It is worth noting that the doping of sulfur in g-C_3_N_4_ can be challenging to quantify accurately, especially when the doping level is low. Doping sulfur with thiourea as a precursor while synthesizing graphitic carbon nitride nanosheets has a relatively low amount of doping compared to other methods or after synthesizing graphitic carbon nitride nanosheets [21]. Also, Fig. 4 a, b, c, d, e and f show that the constituent elements of ZnSnO_3_@S-doped g-C_3_N_4_, which include N, O, S, Sn, C, and Zn respectively, are uniformly dispersed.

**Fig. 5:**
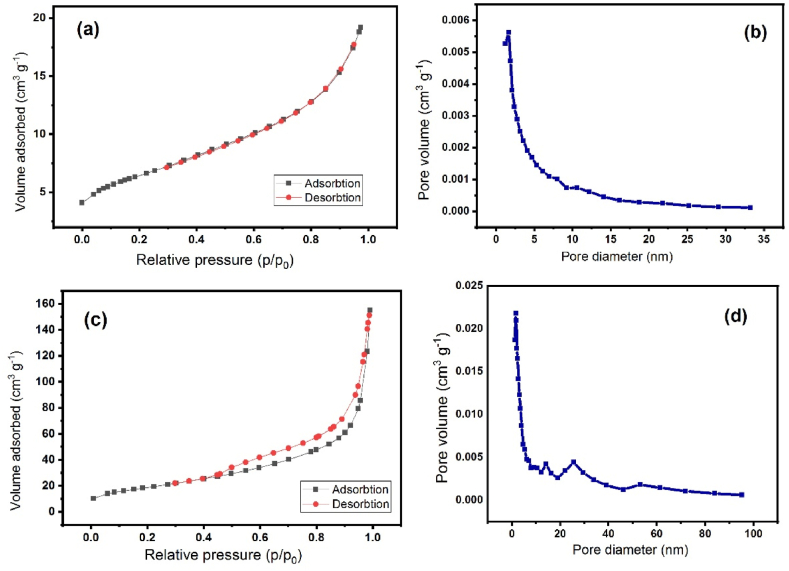
**(a)** BET analysis of S-doped g-C_3_N_4_, **(b)**BJH analysis of S-doped g-C_3_N_4_, **(c)** BET analysis of ZnSnO_3_@S-doped g-C_3_N_4_,and **(d)** BJH analysis of ZnSnO_3_@S-doped g-C_3_N_4_.

Specific surface areas and porosities of the nanocomposite were obtained by BET analysis through the absorption and desorption of nitrogen gas (Fig. 5 a,c). The surface area of nanocomposite ZnSnO_3_@S-doped g-C_3_N_4_ is 68.406 m^2^/g. The holes are 13.947 nm in size and have a volume of 0.2385 cm^3^/g.

BET analysis can also be used to determine the size of the holes. Pore size distribution diagrams are known as BJHs. As a result of this analysis, a pore size distribution can be calculated. Fig. 5(b and d) shows the size distribution of nanocomposite pores between 1 and 100 nm. The average volume of holes based on this method is about 0.236 cm^3^/g.

### 3.2 Parameter optimization

Many parameters affect the degradation of DB1 dye using the photocatalytic ozonation process. The effect of parameters such as different photocatalyst percentages, pH, temperature, time of the process, photocatalyst amount, initial concentration of DB1 dye, and the rate of ozone flow was optimized.

#### 3.2.1 The effect of different photocatalyst percentages

**Fig. 6:**
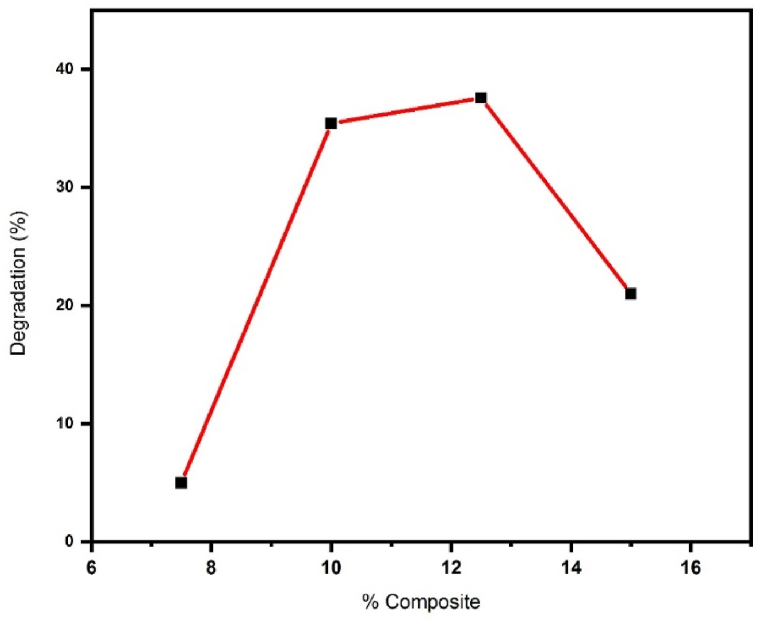
Degradation of DB1 dye by different photocatalyst percentages.

At this stage, the nanocomposite was synthesized in proportions of 7.5 %, 10 %, 12.5 %, and 15 % ZnSnO_3_. These nanocomposites comprised 7.5 %, 10 %, 12.5 %, and 15 % ZnSnO_3_, respectively. In addition, they contained 92.5 %, 90 %, 87.5 %, and 85 % S-doped g-C_3_N_4_. According to the results (Fig. 6), the nanocomposite with 12.5 % ZnSnO_3_ exhibited the highest dye degradation efficiency. Hence, 12.5 % ZnSnO_3_@S-doped g-C_3_N_4_ was selected for further research [23].

#### 3.2.2 The effect of pH

**Fig. 7:**
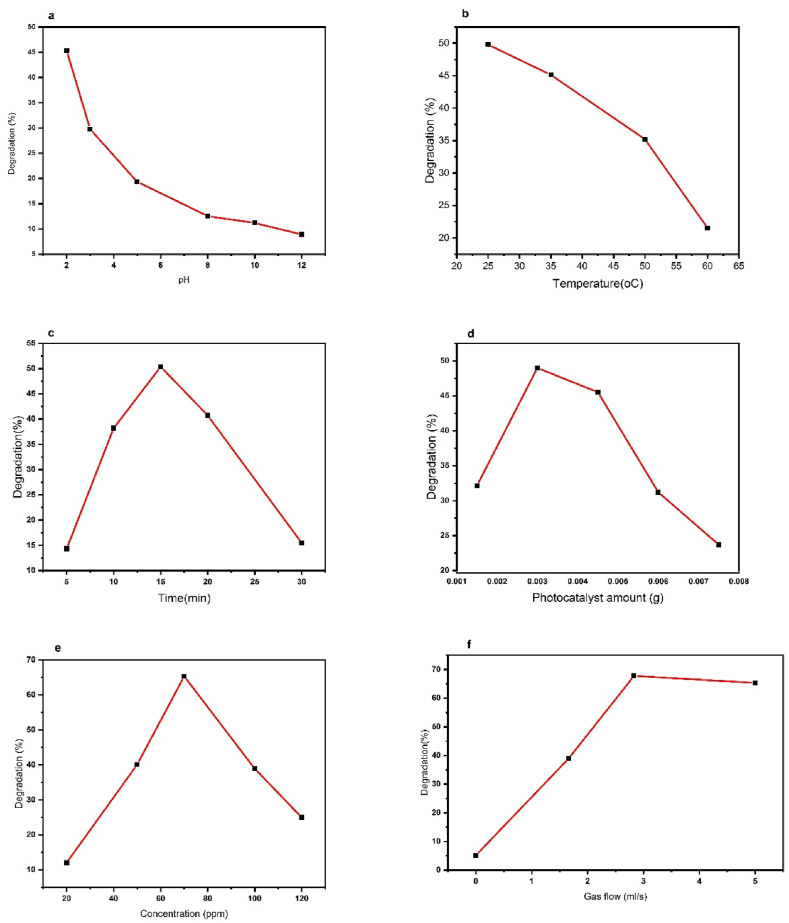
Optimization results: **(a)** pH, (**b)** Temperature (°C), **(c)** Time (min), **(d)** Photocatalyst amount (g), **(e)** Concentration of DB1 dye(ppm), and **(f)** ozone flow rate (ml/s).

To evaluate the effect of the pH of the DB1 dye photocatalytic degradation, the pH values were checked from 2 to 12 (Fig. 7 a). Dye degradation is strongly influenced by pH, in more acidic pH leads to more degradation. Acidic conditions have been found to enhance the photodegradation of DB1 through the photocatalysis process. This is because the presence of protons in acidic conditions can generate hydroxyl radicals (·OH), which are highly reactive and can attack the molecular bonds of the dye, leading to its degradation [30]. In contrast, basic conditions can lead to the formation of stable dye-metal complexes caused by hydroxide ions (OH⁻), which can inhibit degradation and reduce the effectiveness of photodegradation processes [31]. Thus, pH = 2 was chosen as the best pH and was used in the next steps. Utilizing acidic pH in other research has demonstrated that more acidic pH values enhance the mineralization rate in the photocatalytic ozonation process ([12,[32], [33], [34]]). While our study revealed the highest degradation efficiency at pH 2, we recognize that this highly acidic condition may not be directly applicable to large-scale industrial operations in wastewater treatment. It is important to consider the practical considerations and limitations when implementing such extreme pH conditions in real-world scenarios. The purpose of examining the pH range in our study was to gain a comprehensive understanding of the catalyst's performance under varying conditions. We acknowledge that the primary focus of our investigation was to evaluate the effectiveness of the photocatalytic ozonation process, specifically highlighting the contributions of ozone and the ZnSnO_3_@S-doped g-C_3_N_4_ catalyst. To address the practical applicability of our findings, it is crucial to emphasize that the highest degradation efficiency observed at pH 2 serves as a reference point to demonstrate the exceptional catalytic activity of the ZnSnO_3_@S-doped g-C_3_N_4_ catalyst. However, we also conducted experiments at pH 3 and 5, which are more relevant to real-world applications. The results from these experiments indicated significant dye removal efficiency within the slightly acidic range, suggesting potential feasibility for industrial-scale wastewater treatment.

#### 3.2.3 Temperature's effect

Experiments were conducted on the effect of temperatures on the degradation of DB1 dye as shown in Fig. 7 b. As the temperature increased, the degradation percentage gradually decreased. It may be due to a decrease in the solubility of ozone gas as the temperature rises. This is because, at higher temperatures, ozone gas has a lower solubility in water. As a result, less ozone dissolves in the water, which can lead to a decrease in the efficiency of the degradation process that depends on the generation of hydroxyl radicals from ozone [35]. In this case, the 25 °C temperature of the test environment was chosen as the most suitable temperature. In the current study, testing at temperatures lower than the ambient temperature (25 °C) was not performed due to the challenges associated with reducing temperatures in the textile industry.

#### 3.2.4 Effect of time

The reaction was carried out at different times to evaluate the impact of the duration of the degradation (Fig. 7 c). By increasing the time from 5 to 15 min, enough time was provided for hydroxyl radical production and subsequent degradation, after which the efficiency decreased. Possibly, this occurs because some of the dye that had been absorbed during the adsorption in the dark stage was released after 15 min by the catalyst particles. This allows it to reach equilibrium with the solution. Furthermore, it is possible that the photocatalyst's active surface sites may become saturated by oxidation products or other substances present in the solution, leading to a decrease in the degradation efficiency [36]. As the optimum time passes, the dye may desorb from the surface of the photocatalyst and reduce the efficiency of the degradation rate [37].

#### 3.2.5 The effect of catalyst dosage

The effect of the amount of ZnSnO_3_@S-doped g-C_3_N_4_ on the degradation of DB1 is shown in Fig. 7 d. The percentage of degradation increased by increasing the photocatalyst amount up to 0.003 g. This may be due to an increase in active sites on the photocatalyst surface. This in turn causes the production of free hydroxyl radicals and the degradation of dye molecules. At higher values, the percentage of degradation decreases over time. It could be caused by a decrease in the transparency of the solution and insufficient light, resulting in ineffective photocatalysis. Furthermore, the interaction and accumulation of photocatalyst particles at high dosages. Hence, 0.003 g was chosen as the optimal value.

#### 3.2.6 Effect of initial concentration

To produce photocatalytic activity, free hydroxyl radicals must reach the surface of the photocatalyst. The concentrations of solutions ranged from 20 ppm to 120 ppm, as shown in Fig. 7 e. Based on the results, increasing the concentration probably increases the dye's interaction with the photocatalyst and improves its efficiency. High concentrations, however, cover the photocatalyst's active sites with dye molecules. Consequently, it is not able to receive light. Thus, 70 ppm was chosen as the optimal concentration.

#### 3.2.7 The effect of the flow of ozone gas

Ozone gas was injected into the DB1 dye solution at different flow rates (Fig. 7 f.). As can be seen, in the absence of ozone (gas flow in the solution is equal to zero ml/s), the lowest degradation is observed. Possibly, electrons produced in the photocatalyst's conduction band cannot find acceptors in the solution, so they recombine with the holes. By injecting ozone gas, the electrons produced in the conduction band of the semiconductor are reacted with ozone. Finally, it increases the production of hydroxyl radicals during the photocatalytic degradation process. In turn, this increases the dye degradation percentage. As the flow rate of ozone gas in the solution rises, the rate of electron-hole recombination decreases, and instead, as seen in Fig. 7 f, when the ozone gas flow rate reaches 2.83 mL/s, maximum photocatalytic degradation occurs. After this amount, the increase in ozone gas flow showed almost a constant effect on the degradation process, which is probably due to the fact that the active sites on the surface of the photocatalyst are stable and produce a constant amount of electron-holes. When ozone (O_3_) is irradiated with light, it decomposes into partial oxygen (O_2_) and oxygen radicals (•O). Both O_3_ and generated O_2_ act as electron acceptors to generate (•O_3_) and (•O_2_) that react with water molecules to produce hydroxyl radicals, which are strong oxidizers [38]. The generated •OH, along with the free electrons produced by the photocatalyst, facilitate the oxidation of organic pollutants in the solution [39]. Further, ozone can directly react with organic compounds and produce hydrogen peroxide, which can then react with (•O_2_) and produce hydroxyl radicals [[40], [41], [42]].

### 3.3 Recyclability of photocatalysts

**Fig. 8:**
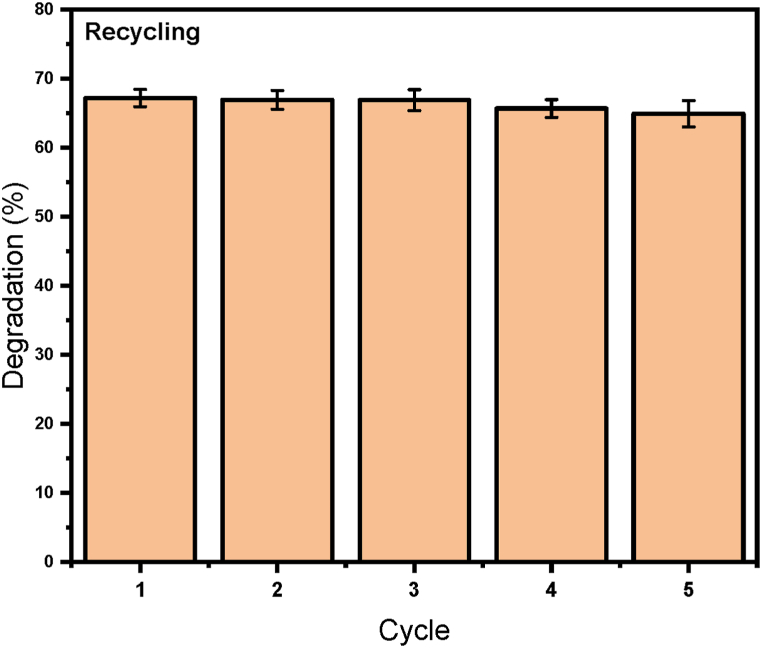
Result of photocatalyst stability after 5 times.

In Fig. 8, the ZnSnO_3_@S-doped g-C_3_N_4_ photocatalyst shows acceptable stability for reuse during photocatalytic ozonation degradation of DB1 dye up to 5 times under optimal conditions without a significant decrease of degradation recovery.

### 3.4 Real sample

To better simulate real-world conditions, the experiment was performed under optimal conditions using a dye solution that was prepared from wastewater comprising of fat, detergent, and dust. The obtained results (Table 1) reveal that the degradation of DB1 dye was significantly accomplished.

**Table 1:** Real sample result.

Sample	Degradation (%)	DB1(ppm)
Distilled water	67.74	70
Real sample	66.2	70

### 3.5 TOC analysis for evaluation of degradation performance

TOC (Total Organic Carbon) is one of the most relevant indicators of wastewater quality. The purification process attempts to reduce organic carbon levels to a standard level. To check the efficiency of ZnSnO_3_@S-doped g-C_3_N_4_ in the degradation of DB1 dye, TOC analysis was performed in 4 stages (Table 2). According to the following equation (2), total organic carbon was calculated:

(2) %TOC = [(B-A)- (C/D)] × 100

**Table 2:** TOC data in different conditions.

Sample	C(ppm)	Condition
A	3	Photocatalyst (at light)
B	5.9	Photocatalyst + DB1(light)
C	1.8	Photocatalyst + DB1(dark)
D	2.6	Stock

Calculations based on the TOC data (Table 2) show that 42 % of the solution consists of organic carbon compounds (TOC). The remaining 58 % was converted to inorganic carbon compounds. This is consistent with the spectrophotometric analysis which showed that the photocatalytic process mineralized 85 % of sample DB1. This is confirmation that photocatalytic ozonation is a highly effective technique for the mineralization of organic compounds.

### 3.6 Kinetics of DB1 dye degradation

**Fig. 9:**
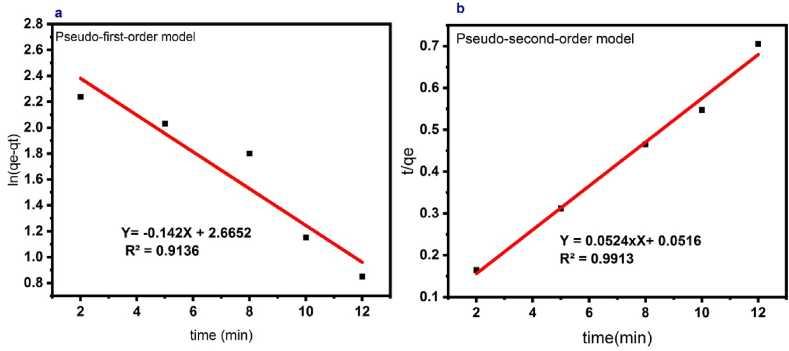
Kinetics model for DB1 decolorization at different initial concentrations. **(a)** First-order model, and **(b)** Second-order model.

To check the speed of the DB1 dye degradation through the photocatalytic ozonation process, the experiment was carried out in optimal conditions in the period of 2–12 min Fig. 9 (a and b). Then the following two equations were used, which are pseudo-first-order (equation (3)) and pseudo-second-order (equation (4)), respectively:

(3) $$\text{Log}\;\left(Q_{e}‐Q_{t}\right)=\log\;Q_{e}‐\;k1\;t\;/2.303$$

(4) $$T/q_{t}=1/\;k_{2}\;{\text{qe}}_{2}+t\;/q_{e}$$

Where Q_e_ and Q_t_ are the degraded sample's equilibrium values at t(min). K_1_(1/min) is the pseudo-first-order rate constant and K_2_ (g/mg.min) is the pseudo-second-order rate constant [43].

Based on the results (Table 3), this process corresponds to the pseudo-second-order model due to the more favorable correlation coefficient (R^2^).

**Table 3:** Kinetics models parameters for DB1 dye degradation.

Kinetic model	R^2^	K	q_e_ (mg/g)
First-order	0.943	0.810	1.25
Second-order	0.9913	2.49	8.39

### 3.7 Absorption isotherms of DB1 dye

The Langmuir and Freundlich isotherm models are commonly used to explain adsorbent-adsorbate interactions. Adsorption occurs on one layer in the Langmuir isotherm model (equation (5)) but on heterogeneous surfaces in the Freundlich isotherm model (equation (6)).

(5) $$C_{e}/Q_{e}=C_{e}/Q_{\max}+1/k_{L}\;Q_{\max}$$

(6) $$\mathrm{Log}\;\left(Q_{e}\right)=\log\;C_{e}/n+\log\;k_{F}$$

C_e_ (mg/l) is the equilibrium concentration of the sample, Q_e_ (mg/g) is the sample's absorption capacity in equilibrium, Q _max_(mg/g) is the maximum absorption capacity of the absorbent on the adsorbent, K_L_ (L/mg) is the Langmuir adsorption isothermal constant and K_F_ (mg/g) (mg/L)n is the Freundlich adsorption isothermal constant [43].

**Fig. 10:**
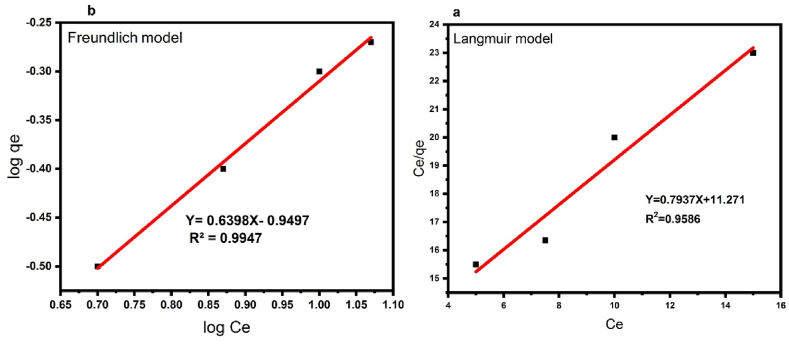
**(a)** Langmuir and **(b)** Freundlich models for DB1 decolorization.

Langmuir and Freundlich isotherms were drawn in Fig. 10 (a and b), and the data related to them were calculated according to equations (3), (4)). Also, the data related to them were summarized in Table 4. In comparing the correlation coefficients (R^2^) between these two types of isotherms, it was found that the Freundlich model is more advantageous as absorption occurs chemically and in multiple layers.

**Table 4:** Adsorption isotherm model parameters for DB1 decolorization.

Adsorption isotherm	Langmuir			Ferundlich		
Constant Amount	R^2^	K_L_	q_m_	R^2^	K_F_	n
	0.9586	0.094	0.93	0.9947	0.133	1.823

## 4 Conclusions

ZnSnO_3_@S-doped g-C_3_N_4_ nano photocatalyst has been synthesized by hydrothermal process. The BET analysis revealed that it has a fifth type of isotherm, which is seen in materials with mesopores [44]. Then it tested for degradation of DB1 dye via photocatalytic ozonation. Ozone gas and pH play a significant role in the degradation of this dye. This catalyst degraded DB1 dye (70 ppm) in 15 min at 25 °C, pH 2, and 2.83 ml/s of ozone flow (regardless of absorption share). Furthermore, the classical analysis revealed that the model is pseudo-second order.

We recognize that further investigations are necessary to explore the catalyst's performance across a broader pH spectrum, including neutral and slightly acidic conditions that align with practical requirements. In consideration of these limitations, our study underscores the importance of optimizing the photocatalytic ozonation process by carefully adjusting the pH to a more practical and operationally feasible range. This optimization process will ensure the successful implementation of the ZnSnO_3_@S-doped g-C_3_N_4_ catalyst in large-scale wastewater treatment facilities, addressing the challenges faced by the textile industry and contributing to the development of efficient and sustainable treatment methods. Despite the many advantages of g-C_3_N_4_, S-doped g-C_3_N_4_, and ZnSnO_3_ photocatalysis, they have a high electron-hole recombination rate [16]. Therefore, the use of ozone in combination with photocatalysts will enhance efficiency [12]. On the other hand, the generated holes can react with water molecules under acidic conditions to produce hydroxyl radicals, which oxidize the pollutant molecules [38]. Additionally, other oxidative species such as •O_2_^−^ or •O_3_^−^ can increase the degradation of pollutants in an aqueous environment [45,46]. ZnSnO_3_@S-doped g-C_3_N_4_ decomposes the harmful molecules of dye into smaller and less harmful molecules after a short time. Additionally, this catalyst is stable enough to be recycled and reused. In light of this, it can be used to solve problems associated with the effluent of the textile industry.

The present research can be compared with other research conducted on DB1 dye, which is reported in Table 5, to conclude that ZnSnO_3_@S-doped g-C_3_N_4_ nanocomposite is a suitable method for removing this dye from the environment and can remove a suitable percentage of it in less time than other methods.

**Table 5:** comparison of different methods for removal of DB1 dye.

Methods	DB1 Concentration (ppm)	Percentage of degradation/removal	Dosage of catalyst (mg L^−1^)	Time (min)	Ref.
Photocatalysis with C_5_N_4_ irradiated	50	100	100	60	[47]
AOPs using pulsed corona discharge from water	10	75–80	200	60	[48]
Photocatalytic degradation with biogenic copper	25–100	88.42	100	3000	[49]
Biotransformation by Marinobacter sp.	100	100	–	3600	[50]
Photocatalysis with TiO_2_ and ZnO	50	100	–	50	[51]
fungus Aspergillus terreus GS28	100	98.4	–	10080	[52]
This work	50	(Removal + Degradation) 100	200	15	This work

## Data availability statement

Data used in this research will be made available upon reasonable request. However, all the data used is in the manuscript.

## CRediT authorship contribution statement

**Elaheh Bahadorirad:** Writing – original draft, Visualization, Software, Project administration, Methodology, Investigation, Formal analysis, Data curation. **Shahab Maghsoudi:** Writing – review & editing, Visualization, Validation, Supervision, Software, Resources, Project administration, Methodology, Investigation, Funding acquisition, Formal analysis, Data curation, Conceptualization. **Elham Jalali:** Writing – review & editing, Visualization, Validation, Software, Resources, Project administration, Methodology, Investigation, Formal analysis, Data curation, Conceptualization.

## Declaration of competing interest

The authors declare that they have no known competing financial interests or personal relationships that could have appeared to influence the work reported in this paper.
