# Supplementary material for: Preparation of nanostructured photocatalyst ZnSnO3@S-doped g-C3N4 and its use in DB1 dye degradation through photocatalytic ozonation process
Source: Heliyon. 2024 Feb 1;10(3):e25451. doi: 10.1016/j.heliyon.2024.e25451 (PMC10861992; doi:10.1016/j.heliyon.2024.e25451)
Supplement: Multimedia component 1 [file mmc1.docx]

**Prime novelty statement**

In this study, the decolorization of DB1 dye, one of the most commonly used dyes in the textile industry, was attempted using the photocatalytic ozonation (PCO) process. Due to its resistance, DB1 dye is difficult to remove from wastewater in these sectors. To this end, ZnSnO_3_@S-doped g-C_3_N_4_ nano-photocatalyst was prepared by a simple hydrothermal method and then, for the first time, a light/O_3_/ZnSnO_3_@S-doped g-C_3_N_4_ system was used for the degradation of this dye. The results were shown that this catalyst is stable enough to be suitable for recycling and re-use. As a result, it could be a solution to the textile industry's wastewater problems. Not only is photocatalytic ozonation often more cost-effective than photocatalysis, but synergistic effects have been observed with the combination of ZnSnO_3_@S-doped g-C_3_N_4_ and ozone.
